# Cell Responses to Extracellular α-Synuclein

**DOI:** 10.3390/molecules24020305

**Published:** 2019-01-15

**Authors:** Alexei A. Surguchev, Fatemeh Nouri Emamzadeh, Andrei Surguchov

**Affiliations:** 1 Department of Surgery, Section of Otolaryngology, Yale School of Medicine, Yale University, New Haven, CT 06520, USA; alexei.surguchev@yale.edu; 2 Division of Biomedical and Life Sciences, Faculty of Health and Medicine, University of Lancaster, Lancaster LA1 4AY, UK; Fatemeh.NouriEmamzadeh@fda.hhs.gov; 3 Department of Neurology, University of Kansas Medical Center, Kansas City, KS 66160, USA

**Keywords:** synucleins, protein misfolding, neurodegeneration, Parkinson’s disease, integrins, glaucoma, matrix metalloproteinases, conformational diseases

## Abstract

Synucleins are small naturally unfolded proteins involved in neurodegenerative diseases and cancer. The family contains three members: α-, β-, and γ-synuclein. α-Synuclein is the most thoroughly investigated because of its close association with Parkinson’s disease (PD), dementia with Lewy bodies and multiple system atrophy. Until recently, the synuclein’s research was mainly focused on their intracellular forms. However, new studies highlighted the important role of extracellular synucleins. Extracellular forms of synucleins propagate between various types of cells, bind to cell surface receptors and transmit signals, regulating numerous intracellular processes. Here we give an update of the latest results about the mechanisms of action of extracellular synucleins, their binding to cell surface receptors, effect on biochemical pathways and the role in neurodegeneration and neuroinflammation.

Synucleins are a family of proteins containing three members, α-, β-, and γ-synuclein implicated in neurodegenerative diseases and cancer. Synucleins are unique to vertebrates and primarily expressed in neural tissue and in certain tumors. Amino acid sequences of all three members of the family have in common a highly conserved alpha-helical lipid-binding domain and several repeats with a general sequence KTKEGV. Despite their overall sequence similarity, the members of the family exhibit differences in their biochemical properties, playing a variety of roles inside and outside of the cell. Since the discovery of the first member of the family 30 years ago synucleins attract continuous attention of researchers because of their unusual properties and association with human diseases [1]. Members of the synuclein family are readily secreted [2] and circulate between cells. Several hypotheses including endocytosis, exosomes [2,3,4,5], and tunneling nanotube formation [6] were generated to explain synuclein secretion. However, none of them was completely satisfactory and did not explain all the unusual properties of α-synuclein. α-Synuclein may be released into extracellular space as a result of oxidative stress [7] and other stress conditions. Interestingly, synucleins do not contain signal peptides at the N-termini and therefore use unconventional mechanism of secretion [8,9]. Moreover, α-synuclein spreads out in a prion-like manner between neurons and other cell types, contributing to the dissemination of the pathology.

Synucleins are relatively small proteins (127–140 amino acids for human proteins), but the tiny size cannot explain their secretion, cell-to-cell-spreading and propagation, since aggregated forms of the protein are also circulating between cells. These forms include large misfolded preformed fibrils (PFF) of α-synuclein with 200 nm in size or bigger [10]. This enigmatic mechanism draws a lot of attention from researchers, since these proteins not only initiate pathology, but also contribute to its propagation. As a result, they are attractive targets for the pharmacological interventions for neurodegenerative diseases. Another unresolved question concerning α-synuclein biochemistry is what is a trigger that initiates the conversion of this protein from its normal physiological functions to a pathological role, associated with neurotoxicity and prion-like properties? Molecular mechanisms underlying α-synuclein secretion, intercellular propagation, as well as its ability to acquire prion-like properties and accompanying pathological functions remain obscure. In recent years, the focus of synuclein’s research is shifting from intracellular to extracellular forms of these proteins and their impact on intracellular processes of adjacent cells.

A number of recent studies suggests that extracellular α-synuclein acts as a specific ligand for cell surface receptors [11,12,13,14]. Oligomeric α-synuclein binding to cell surface receptors induces the transmission of signal into cells and causes a variety of biochemical and physiological reactions, including Ca^2+^ dysregulation [15], synaptic dysfunction, neurodegeneration, cognitive deficit, etc. α-synuclein has promiscuous partners, and many synuclein-interacting intracellular proteins have been identified before [16]. Recent results point to an important role of α-synuclein binding to cellular surface receptors which transmit signals affecting intracellular processes.

One of such interacting protein is a cellular prion protein (PrPC). α-synuclein in addition to possessing prion-like properties itself [17,18] directly interacts with PrPC [11,12,13]. This cooperation facilitates the transfer of α-synuclein between cells [11]. Furthermore, such interaction causes synaptic dysfunction via a signaling cascade acting through phosphorylation of Fyn kinase and activation of the N-methyl-D-aspartate receptor [11,12,13,14]. Apparently, α-synuclein and PrPC do not form a tight complex, but are involved in short-term transitory interaction that alters α-synuclein conformation and properties. An important consequence of α-synuclein-PrPC binding is the induction of cofilin/actin rods formation [19], changing actin dynamics and resulting in rearrangements of cytoskeleton (Figure 1). Cofilin-actin bundles or rods formed in axons and dendrites of stressed neurons may cause synaptic dysfunction and mediate cognitive deficits in dementias [19]. Interestingly, another member of the synuclein family, γ-synuclein, is colocalized with cofilin/actin rods located near the nucleus. The number of these structures increases after traumatic brain injury [20]. The effect of ligand binding to cell surface receptors and ion channels inducing rearrangement of actin cytoskeleton is a frequent cause of neurodegeneration [21]. Extracellular α-synuclein also causes microtubule destabilization via GSK-3β-dependent tau phosphorylation [22].

Another cell surface receptor which assists in transmission of misfolded α-synuclein (more exactly misfolded preformed fibrils or PFF of α-synuclein) from neuron to other cells and takes part in signal transduction is a product of lymphocyte-activation gene 3 (LAG3) (former CD223, cluster of differentiation 223) [26]. LAG3 is a member of immunoglobulin superfamily molecule involved in immunoregulation [27]. It is an immune checkpoint receptor possessing various biologic effects on T cell function. The interaction between α-synuclein-PFF with LAG3 provides a new target for the therapeutic interventions with potential to reduce the progression of PD and related α-synucleinopathies. Interestingly, LAG3 binds misfolded α-synuclein built-in in PFF with high selectivity (dissociation constant 77 nM) but does not interact with monomeric α-synuclein. Similar specificity to aggregated, but not monomeric α-synuclein is described for toll-like receptor-2 (TLR2) and transmembrane ion channels receptor P2X7 [28]. The binding induces a concentration-dependent microglial glutamate release and activation of the cystine/glutamate antiporter system Xc [31]. The results of a recent study demonstrate that binding of extracellular α-synuclein to P2X7 receptor-pannexin induces ATP release in neuroblastoma SH-SY5Y cells [32]. Recently, an interaction of synucleins with another protein involved in immunoregulation-CD11b integrin (the α-chain of integrin αMβ2) was described [29]. One more type of receptor that modulate α-synuclein aggregation and toxicity in both nerve cells and microglia is adenosine A2AR heteroreceptor complex [30]. These recent results propose that antibodies against synuclein’s receptors are promising tools for immunotherapy and their application may be considered as a potential therapeutic intervention for synucleinopathies [23].

Remarkably, LAG3 complex with misfolded synuclein is colocalized with small Ras-like GTPases (Rabs), including Rab5a and Rab7, which are mediators of the vesicle recycling and protein traffic [34,35]. As a result, LAG3 in cooperation with Rabs plays an essential role in α-synuclein-PFF endocytosis, cell-to-cell transmission, and internalization of pathologic α-synuclein. Furthermore, since LAG3 is a major player in the immune system, its interaction with misfolded α-synuclein may affect downstream signaling [26]. In addition to Rab5a and Rab7, other small Ras-like GTPases, including Rab3A, Rab5, and Rab8 are associated with aggregated α-synuclein. This data indicates that α-synuclein aggregates have a tendency to sequester Rab proteins [34,35]. Moreover, Rabs-α-synuclein interaction may also influence α-synuclein processing, clearance, spreading, and aggregation [36].

Binding of α-synuclein to cell surface receptors may cause pathological changes through various mechanisms. According to one of them, α-synuclein oligomers released from neuronal cells induce proinflammatory responses from microglia. These responses are mediated by the activation of Toll-like receptor 2 (TLR2) signaling, cytokine receptor signaling and other immune receptor signaling pathways producing various proinflammatory cytokines and chemokines. On the next step, actin cytoskeleton rearrangement pathways and cell migration are activated, while TLR signaling and cytokine and chemokine production are continued [37]. The importance of TLR2 receptor in this process is validated by experiments with its deletion, which resulted in elimination of cytokine/chemokine gene induction by α-synuclein. Thus, α-synuclein released from cells is an endogenous agonist for TLR2 through which microglia are activated and become neurotoxic. This study shows that α-synuclein oligomers are inducers of inflammatory innate immunity in the nervous system [37].

Another recent study demonstrates that α-synuclein-induced microglial activation may be processed via an alternative pathway omitting TLR2) signaling. This mechanism is carried out via CD11b, the α chain of integrin αMβ2 [29,38]. The activation of microglial NADPH oxidase (NOX2) induced by α-synuclein is a well-known mechanism implicated in Parkinson’s disease (PD) and other synucleinopathies. Recent finding show that integrin CD11b mediates α-synuclein-induced NOX2 activation through a RhoA-dependent pathway. These results suggest a new mechanistic insight and also point to a novel potential therapeutic synucleinopathies [29].

As shown above, synucleins modulate many intracellular processes and interact with a plethora of proteins, affecting signaling pathways [11,12,13,14,16,23,26,29,32]. What structural elements of these proteins ensure their binding diversity and involvement in many cellular functions? Being intrinsically disordered or natively unfolded proteins, synucleins lack an ordered three-dimensional structure and do not autonomously fold up into a unique stable conformation. Unfolded α-synuclein may represent an overlay of at least 50 various structures taken from the protein ensemble database (PyMOL, Schrödinger, Inc. New York, NY, USA) [39]. Such flexible protein conformation and ability to adopt multiple 3D structures have certain physiological advantages. In contrast to proteins with stable secondary and tertiary structure, synucleins possessing flexible conformation can easily alter its 3D structure in response to changing environmental stimuli: ionic strength, pH, binding of small molecules, etc., exposing its hidden domains. Such structural transition allows interaction with specific protein partners to these exposed parts, ensuring binding specificity in response to altered milieu. As a result synucleins may serve as sensors transmitting information in response to changing conditions.

Taking into consideration an important regulatory role of α-synuclein, several attempts have been made to find the way to specifically modify its properties by mutations, post translational modifications (PTMs), interaction with other proteins or compounds. One approach is to modify synuclein’s secondary and tertiary structure by interaction with β-wrapins—genetically engineered binding proteins. β-wrapins are artificial proteins which stabilize the β-hairpin conformations of α-synuclein and other amyloidogenic proteins and inhibit their aggregation and toxicity [33,40]. The exact design of β-wrapins may be optimized for specific pathology by computational methods, molecular dynamics simulations, and free energy calculations. This approach presents a promising therapeutic strategy for inhibition the aggregation and toxicity of amyloidogenic proteins.

In addition to artificial proteins, graphene based nanomaterials GQDs (graphene quantum dots) also could modulate synuclein properties. Graphene are composed of carbon atoms arranged in a hexagonal lattice representing flat polycyclic aromatic hydrocarbons [41]. Graphene based nanomaterials GQDs (graphene quantum dots) and graphene oxide quantum dots (GOQDs) are composed of carbon atoms arranged in a hexagonal lattice representing flat polycyclic aromatic hydrocarbons [42,43]. The development of split-luciferase complementation test may become a base for the generation of bioluminescence biosensors to monitor oligomerization of α-synuclein inside the cells [42].

GQDs inhibit α-synuclein fibrilization, interact with α-synuclein PFFs, induce interaction-coupled unfolding and could dissociate them. Importantly, GQDs can penetrate through brain blood barrier and therefore are promising candidates for pharmacological interventions for synucleinopathies [43]. The development of methods of synuclein’s handling in vitro or in biological systems with using these man-made materials may open a new direction in the development of cure for Parkinson’s disease.

Recent findings of new α-synuclein interacting proteins, cell surface receptors, and engineered materials offer a new explanations of some unusual behavior of α-synuclein inside and outside of a cell. They point to new potential drug targets for synucleinopathies treatment and indicate new connections of α-synuclein with components of immunosystem. Therapeutic intervention may be directed not only to disease-causing molecules, but also on their cell surface receptors and downstream signaling cascades in order to prevent or delay the pathological changes. However, the development of new methods of treatment directed to α-synuclein receptors and to downstream signaling cascades should be implemented with caution taking into consideration the pleiotrophic effects of α-synuclein.

## Figures and Tables

**Figure 1:**
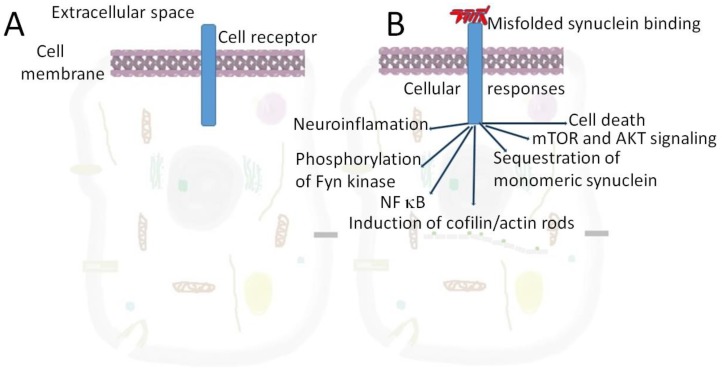
The effect of extracellular α-synuclein binding to cellular receptor on neuronal or glial cells. Various proteins may fulfill the role of such receptors, including PrPC, LAG3, neurexin 1α [17], TLR2 [23], mGluR5 [12], Fc gamma receptor IIb [24] (Table 1) and others. Gangliosides in the lipid rafts can also act as receptors for extracellular α-synuclein [25]. **A**–a cell in the absence of extracellular α-synuclein, **B**–cellular response on α-synuclein binding.

**Table 1:** Cell surface receptors binding extracellular α-synuclein.

Name of the Receptor	Properties	References
N-methyl-D-aspartate receptor	NMDAR—Glutamate ionotropic receptor and ion channel in nerve cells.	[12]
Lymphocyte-activation gene 3 (LAG3) (CD223)	LAG3—immune checkpoint receptor with diverse biologic effects on T cell function.	[26,27]
TLR2 receptors	TLR2—toll-like receptor 2—a membrane receptor expressed on the cell surface binding extracellular molecules and transmitting signals to the cells of the immune system.	[28]
CD11b integrin (the α-chain of integrin αMβ2)	CD11b—transmembrane receptor facilitating cell-extracellular matrix adhesion.	[29]
Adenosine A2AR heteroreceptor complex	Adenosine receptor, G protein-coupled receptor (GPCR) family which possess seven transmembrane alpha helices, as well as an extracellular N-terminus and an intracellular C-terminus.	[30]
PrPC	PrPC—a cellular prion protein. α-Synuclein directly interacts with PrPC [10,11,12]. This cooperation facilitates the transfer of α-synuclein between cells [10] and induces cofilin/actin rods formation.	[11,12,13]
Neurexin-α	Neurexin-α is a presynaptic protein connecting neurons at the synapse. Located mostly on the presynaptic membrane, contains a single transmembrane domain.	[17]
P2X7	PDX7—purinoceptors for ATP serves as a pattern recognition receptor for extracellular ATP-mediated apoptotic cell death.	[28,31,32]
mGluR5	mGluR5—metabotropic glutamate receptor 5 is a member of the family of G protein-coupled receptors	[12]
Fc gamma receptor IIb	FCGR2B is a low affinity receptor for IgG. Mutation in the gene leads to a lupus phenotype	[24]
Gangliosides in the lipid rafts	Gangliosides in the lipid rafts acts as receptors for extracellular α-synuclein [33]	[25]
